# Molecular and Cellular Effects Induced in *Mytilus galloprovincialis* Treated with Oxytetracycline at Different Temperatures

**DOI:** 10.1371/journal.pone.0128468

**Published:** 2015-06-11

**Authors:** Mohamed Banni, Susanna Sforzini, Silvia Franzellitti, Caterina Oliveri, Aldo Viarengo, Elena Fabbri

**Affiliations:** 1 Department of Sciences and Technological Innovation (DiSIT), University of Piemonte Orientale "A. Avogadro", V.le T. Michel 11, 15121, Alessandria, Italy; 2 Laboratory of Biochemistry and Environmental Toxicology, ISA, Chott-Mariem, 4042, Sousse, Tunisia; 3 Department of Biological, Geological and Environmental Sciences, University of Bologna, via Selmi 3, 40100, Bologna, Italy; 4 Interdepartment Centre for Environmental Science Research, University of Bologna, via S. Alberto 163, 48123, Ravenna, Italy; University of Siena, ITALY

## Abstract

The present study evaluatedthe interactive effects of temperature (16°C and 24°C) and a 4-day treatment with the antibiotic oxytetracycline (OTC) at 1 and 100μg/L on cellular and molecular parameters in the mussel *Mytilus galloprovincialis*. Lysosomal membrane stability (LMS), a sensitive biomarker of impaired health status in this organism, was assessed in the digestive glands. In addition, oxidative stress markers and the expression of mRNAs encoding proteins involved in antioxidant defense (catalase (cat) and glutathione-S-transferase (gst)) and the heat shock response (hsp90, hsp70, and hsp27) were evaluated in the gills, the target tissue of soluble chemicals. Finally, cAMP levels, which represent an important cell signaling pathway related to oxidative stress and the response to temperature challenges, were also determined in the gills. Exposure to heat stress as well as to OTC rendered a decrease in LMS and an increase in malonedialdehyde accumulation (MDA). CAT activity was not significantly modified, whereas GST activity decreased at 24°C. Cat and gst expression levels were reduced in animals kept at 24°C compared to 16°C in the presence or absence of OTC. At 16°C, treatment with OTC caused a significant increase in cat and gst transcript levels. Hsp27 mRNA was significantly up-regulated at all conditions compared to controls at 16°C. cAMP levels were increased at 24°C independent of the presence of OTC. PCA analysis showed that 37.21% and 25.89% of the total variance was explained by temperature and OTC treatment, respectively. Interestingly, a clear interaction was observed in animals exposed to both stressors increasing LMS and MDA accumulation and reducing hsp27 gene expression regulation. These interactions may suggest a risk for the organisms due to temperature increases in contaminated seawaters.

## Introduction

Pharmaceuticals and personal care products (PPCPs) are contaminants of emerging concern in the aquatic environment [1;2], and they are present in all aquatic compartments, including influents and effluents [3,4] from waste water treatment plants (WWTPs), rivers and lakes [5], coastal waters [6], groundwater, and drinking water [7]. Their concentration in aquatic environments is considered to be too low to cause acute effects, but they are continuously released and reported to produce chronic effects [8].

A major concern is the presence of antibiotics, which is associated with the development of resistance mechanisms by bacteria [9], butvertebrate and invertebrate species inhabiting aquatic bodies may also be affected.

Many antibiotics are poorly removed by conventional WWTPs and enter receiving waters; those used in aquaculture are promptly available in the aquatic environment. Tetracyclines have been reported as the predominant antibiotics in seawater (2.11–9.23 ng/L) [10] that may reach concentrations of micrograms per liter in coastal environments near fish farms. Oxytetracycline (OTC) is an antimicrobial extensively used in human and veterinary medicine and all over the world for the treatment of bacterial diseases in aquaculture [11] because of itsbroad-spectrum efficacy in the treatment of infections caused bymicroorganisms. OTC inhibits protein synthesis by preventing the associationof aminoacyl-tRNA with bacterial ribosomes [12]. OTC aquatic toxicity has been assessed using standard tests according to OECD guidelines, and effects were found on marine bacteria, green algae, duckweed,crustaceans [11], cyanobacteria, daphnids, and activated sludge [13] at concentrations of milligram per liter. OTC also induces oxidative stress in algal cells at similar concentrations [14].

Effects by aquatic contaminants, including pharmaceutical residues, may be exacerbated by the increasing temperatures associated with global change [15]. Ocean water temperatures are predicted to increase by at least 1.1°C during this century, and increases of up to 3–4°C are possible. In intertidal and coastal areas, the degree of local warming and the amplitude of thermal fluctuations may easily exceed the global predicted increases [16].

As temperature influences physiological processes, an interaction between temperature and toxicants can be expected. The toxicity of conventional pollutants to aquatic animals generally increases at higher temperatures [15], often due to a greater induction of free radical production. Temperature may also influence the metabolic rate and feeding activity of aquatic organisms, as well as pollutant kinetics and detoxification mechanisms [17]; therefore, exposure to toxicants at higher water temperatures may potentiate the toxic effects of chemicals [18;19].

At increased temperatures, animals develop a stress response and shift energy from growth and reproduction to the expression of stress response proteins, including heat shock proteins, antioxidant enzymes, and metallothioneins, which act both as antioxidant scavengers and metal chelators [20;21;22]. The heat shock protein response is among the most investigated physiological pathways in response to global changes [23]. Notably,a decrease in thermo-tolerance has been observed in animals exposed to pollutants [24;25].

Recent reports from our laboratories led to the hypothesis that the load of reactive oxygen species (ROS) may be an important factor that increases the energetic costs in mussels exposed to heat stress [25;26]. Moreover, Franzellitti and Fabbri (2013)[27] suggested that the impairment of regulatory pathways triggered by low concentrations of pharmaceuticals may affect the ability of animals to elaborate defense strategies or adapt to stress factors.

The present study evaluated the effects of OTC at different temperatures on some physiological parameters of the mussel *Mytilus galloprovincialis*. These filter-feeding bivalves are model organisms widely used in biomonitoring programs to evaluate the toxic effects of contaminants [28; 29; 30;31].

Mussels were exposedat 16°Cor 24°C in the presence of OTC at two concentrations (1 or 100 μg/L). First, we sought to identify the effects of sublethal OTC concentrations on a sensitive biomarker, lysosomal membrane stability (LMS), in the cells of the digestive gland, the metabolic organ in these organisms. In the gills, the target organ of soluble chemicals, the effects of OTC were evaluated by measuring malonedialdehyde (MDA) accumulation and the activity of catalase (CAT) and glutathione S-transferase (GST) as indicators of the oxidative stress response. Moreover, the expression levels of the following gene products related to heat and oxidative stress were analyzed by quantitative real time qPCR: catalase (cat), glutathione S-transferase (gst), and heat shock proteins 27, 70, and 90 (hsp27, hsp70, and hsp90).

Since large body of evidence supports a relationship between alterations of the cyclic AMP (cAMP) system by different stressors and the concomitant induction of oxidative stress [32], tissue levels of cAMP were also evaluated. Considering the crucial role displayed by cAMP in a variety of physiological functions of mussels including reproduction, metabolic regulation, filtering capabilitie, and stress responses [32], alterations of the cAMP signaling by environmentally induced oxidative stress have the potential to impair relevant physiological functions in invertebrates and jeopardize the fitness of individuals within a natural population.

## Material and Methods

### Animals and treatments

Specimens of *M*. *galloprovincialis* (Lam.)4–5 cm long were collected from the north-western Adriatic Sea coast during the month of October by fisherman of the “Cooperativa Copr.al.mo” (Cesenatico, Italy, within farm locations approved for the direct commercialisation according to the European legislation 91-492-CEE). Mussels were acclimated for 6 days in aquaria containing clean, aerated seawater collected offshore (at a density of 1 animal/L), a period of time sufficient to stabilize at control temperaturethe mussel physiological response [33]. Groups of mussels were then kept in 16 polypropylene plastic vessels (four replicates per treatment) at 16°C or 24°Cand underwent semi-static exposure to 1 μg/L or 100 μg/L OTC for 4 days. Control animals were maintained in seawater at the two experimental temperatureswith no addition of OTC. Seawater at the desired temperature was renewed every day, and OTC was added with a commercial algal preparation (30 mg/animal/day) (Liquifry; Interpret Ltd., Dorking, Surrey, UK). Only females,as determined by microscopic inspection of gonad biopsies, were selected for subsequent analysis to avoid gender-based bias. After treatment, the gills were rapidly removed, frozen in liquid N2, and stored at -80°C for CAT, GST, and MDA analysis. A second set of gills were kept at -20°C in an RNA-preserving solution (RNALater; Sigma-Aldrich) for RT-PCRanalysis. The digestive glands were placed on aluminum chucks and chilled in hexane at -70°C and then utilized for histochemical determination of LMS.

### Lysosomal membrane stability

LMS was evaluated in cryostat sections (10 μm) of five digestive glands as described previously [34]. This cytochemical assay is based on the acid labilization characteristics of latent hydrolase β-N-acetylhexosaminidase (NAH) using naphthol AS-BI-N-acetyl-β-D glucosaminide as a substrate for NAH. Slides were observed at 400× magnification using an inverted microscope (Zeiss Axiovert 100M) connected to a digital camera (Zeiss AxioCam). The obtained pictures were analyzed using an image analysis system (Scion Image freeware). Data are expressed as labilization time (mn).

### MDA content

For each condition, gills from 20 individuals were combined into four pools (one per vessel). Aliquots(0.5–1 g) werehomogenized in 2 volumes of buffer containing Tris HCl (20 mM,pH 7.4) and 0.1% mercaptoethanol. The homogenate was centrifuged at 18,000 × g (4°C) for 20 min. The MDA concentration was determined in the supernatant as described previously [35]. MDA was expressed as μmole/g of fresh tissues.

### Antioxidant enzyme activities

For each condition, gills from 20 individuals were combined into four pools (one per vessel). Aliquots (0.5–1 g) were homogenized in 50mM potassium phosphate buffer (KPB), pH 7.0, containing 0.5mM Na_2_EDTA as previously reported [36]. Enzyme activities were measured using a DU 800 Multisample spectrophotometer (Beckman) at 25°C. Catalase (EC 1.11.1.6) activity was determined by measuring the decrease in absorbance at 240 nm due to the consumption of hydrogen peroxide (55mM H2O2 in 50mM KPB, pH 7.0). The reaction was followed for 2 min. Results were expressed as micromoles hydrogen peroxide transformed per minute and per milligram protein. Glutathione S-transferase (EC 2.5.1.18) activity was estimated by the increase in absorbance at 340 nm due to the conjugation of 1-chloro-2,4-dinitrobenzene (CDNB) with reduced glutathione (GSH). The reaction was followed for 10 min. Results were expressed as nanomoles GSH-CDNB produced per minute and per milligram protein. Protein content was assessed according to the method of Lowry et al.(1951) [37].

### Analysis of mRNA expression

Total RNA was extracted from individual gill pieces (pool of three females) using acid phenol-chloroform precipitation according to Chomczynski and Sacchi(1987) [38] with TRI-Reagent (Sigma-Aldrich). The RNA was further purified by precipitation in the presence of 1.5 M LiCl2, and the quality of each RNA preparation was confirmed by UV spectroscopy and TBE agarose gel electrophoresis in the presence of formamide.

The mRNA abundance of genes encoding CAT, GST,hsp27, hsp70, and hsp90 were evaluated in multiplex Taqman assays as previously described[25;26;39]. Probes and primer pairs (Table 1) were designed using Beacon Designer v3.0 (Premier Biosoft International, Inc.). All primers and dual-labeled Taqman probes were synthesized by MWG-Biotech Gmbh (Germany). cDNA reverse transcribed from 25 ng RNA was amplified in a CFX384 Real-Time PCR detection system (Bio-Rad Laboratories) with iQTM Multiplex Power mix (Bio-Rad Laboratories) according to the manufacturer’s instructions for the triplex protocol. All multiplex combinations accounted for the following dual fluorescence tags: 6-carboxyfluorescein/Black Hole (BH) 1, 6-carboxy-2′,4,4′,5′,7,7′-hexachlorofluorescein/BH1, and Texas Red/BH2. Briefly, the cDNA was amplified in the presence of 1X iQTM Multiplex Power mix, 0.3 μM of each primer, and 0.1 μM of each probe (Table 1) in a final volume of 10 μL. Relative expression data were geometrically normalized to 18S rRNA (L33452), an invariant actin isotype (AJ625116), and ribosomal protein riboL27 (AJ625928) [26], which were selected from a list of genes for whichexpression did not vary over more than 50 conditions,including toxic treatments, life cycle stages, and various tissues. A specific triplex Taqman assay was developed to amplify 0.25 ng of RNA reverse-transcribed to cDNA in the presence of 0.1 μM of each dual-labeled probe (hexachlorofluorescein/BH1 for actin, Texas Red/BH2 for 18S rRNA, and Hex/BH2 for protein riboL27) and 0.1 μM, 0.4μM, and 0.4 μM of forward and reverse primer for 18S rRNA, actin, and proteinriboL27, respectively (Table 1). For all Taqman assays, the thermal protocol was as follows: 30 s at 95°C, followed by 40 cycles of 10 s at 95°C and 20 s at 60°C. qRT-PCR was performed with four biological replicates and three technical replicates.

**Table 1:** Q-PCR primers and Taqman probes.

Gene name	Probe	Sense Primer	Antisense Primer
RiboL27	TGCGCCATTCAGCACAAGAACTACCT	AAGCCATGGGCAAATTTATGAAAA	TTTACAATGACTGCTTTACGACCT
β-Actin	ACGCCAACACCGTCTTGTCTGGTGG	GTGTGATGTCATATCCGTAAGGA	GCTTGGAGCAAGTGCTGTGA
18S	ACCACATCCAAGGAAGGCAGCAGGC	CGGAGAGGAGCATGAGAAAC	CGTGCCAGGAGTGGGTAATTT
cat	ACAGCTTGTCTGCCTGCTCAGCAC	ACAAGGATGGACAGGCATACTAC	AATCACGGATGGCATAATCTGGA
gst	ACGCCTGTGTCCCCAAACAAGTGG	AACTGACCACTTCAAGAATATGCC	AGAAAGTCTGCCATTTACAAAGCT
Hsp 27	CTTGGTGTGCAGTGACAGAGTCCT	TTGTCCCACAGAAGGCTAAGG	ACAGACCGTGGAACTGTAATG
Hsp 70	ACCACACCGAATAGTGAAGATCTGCCA	CCGTCCACACCACCCACC	GTGAGGTTAGCTGACAATGGTGG
Hsp90	ATCAGCTCCAGCTTGAAGAGCCTCCAT	AAGCTGATCTGGTCAATAACCTGG	AACCTACACCAAACTGTCCAATCA

Given are: Gene ID, NCBI gene Identifier; Taqman probe, sense primer and antisense primer sequences. All sequence are given 5′ to 3′. Legend: Ribosomal protein genes ribo-L27 (AJ625928), actin (AJ625116), 18S ribosonal RNA (L33452),cat (AY580271.1), gst (AF527010.1),hsp27 (AJ625244), hsp70 (AJ624049), and hsp90 (AJ625915).

### Cyclic AMP signaling

For each condition, gills from 20 individuals were combined into four pools (one per vessel). Aliquots of approximately200 mg were homogenized with 3 volumes of 6% trichloroacetic acid. After centrifugation (600 × g, 15 min, 4°C), supernatants were washed three times with 3 volumes of water-saturated ether, and the final aqueous extracts were dried under a stream of nitrogen. Samples were assayed for cAMP content using the DetectXTM direct cyclic AMP enzyme immunoassay kit (Arbor Assay, USA) according to the manufacturer’s protocol. Results were expressed as picomole cAMP per gram of fresh tissue (n = 4).

### Statistical analysis

Biomarker data were analyzed using the SigmaStat statistical package. Significant differences between treatment groups were determined using one-way ANOVA followed by the Mann-Whitney U-test. Real time PCR data were analyzed with the REST software [40], which uses a randomization test with pairwise reallocation to assess the statistical significance of the differences in expression between each treatment-exposure group and the reference group. In any case, statistical differences were accepted when p < 0.05. Factor analysis using the principal component analysis (PCA) extraction procedure was performed in the STATISTICA software package (StatSoft) in order to determine whether OTC treatment had significantly different effects at 16°C and 24°C.

## Results

**Fig 1:**
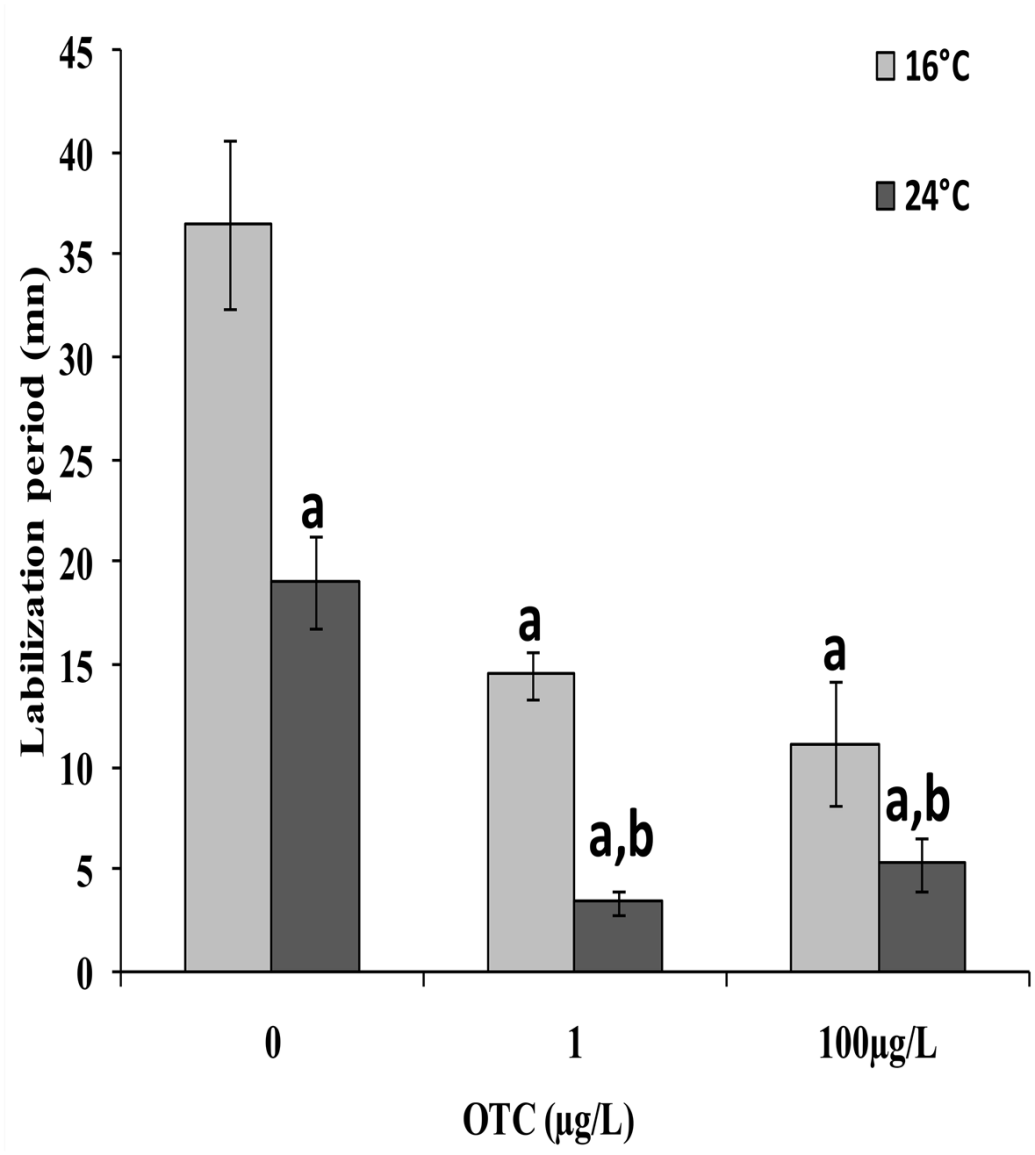
Lysosomal membrane stability (LMS) in digestive gland sections of *M*. *galloprovincialis* individuals after a 4-day treatment with 1 or 100 μgtetracycline at 16° and 24°C. Data are expressed as the Labialization period in mn are represent the mean ± Sd (N = 4). (a: significantly different from reference condition. b: significantly different from OTC-untreated mussels at 24°C (p < 0.05).

The effects of OTC exposure and temperature increase on LMS are reported in Fig 1. At 16°C, a pronounced effect of OTC was detected for the two tested concentrations compared to control mussels. Exposure to heat stress only (24°C) was effective at decreasing LMS, and a further significant decrease in LMS was recorded after treatment with OTC at 24°C. OTC effects at 24°C were significantly greater than those observed at 16°C.

**Fig 2:**
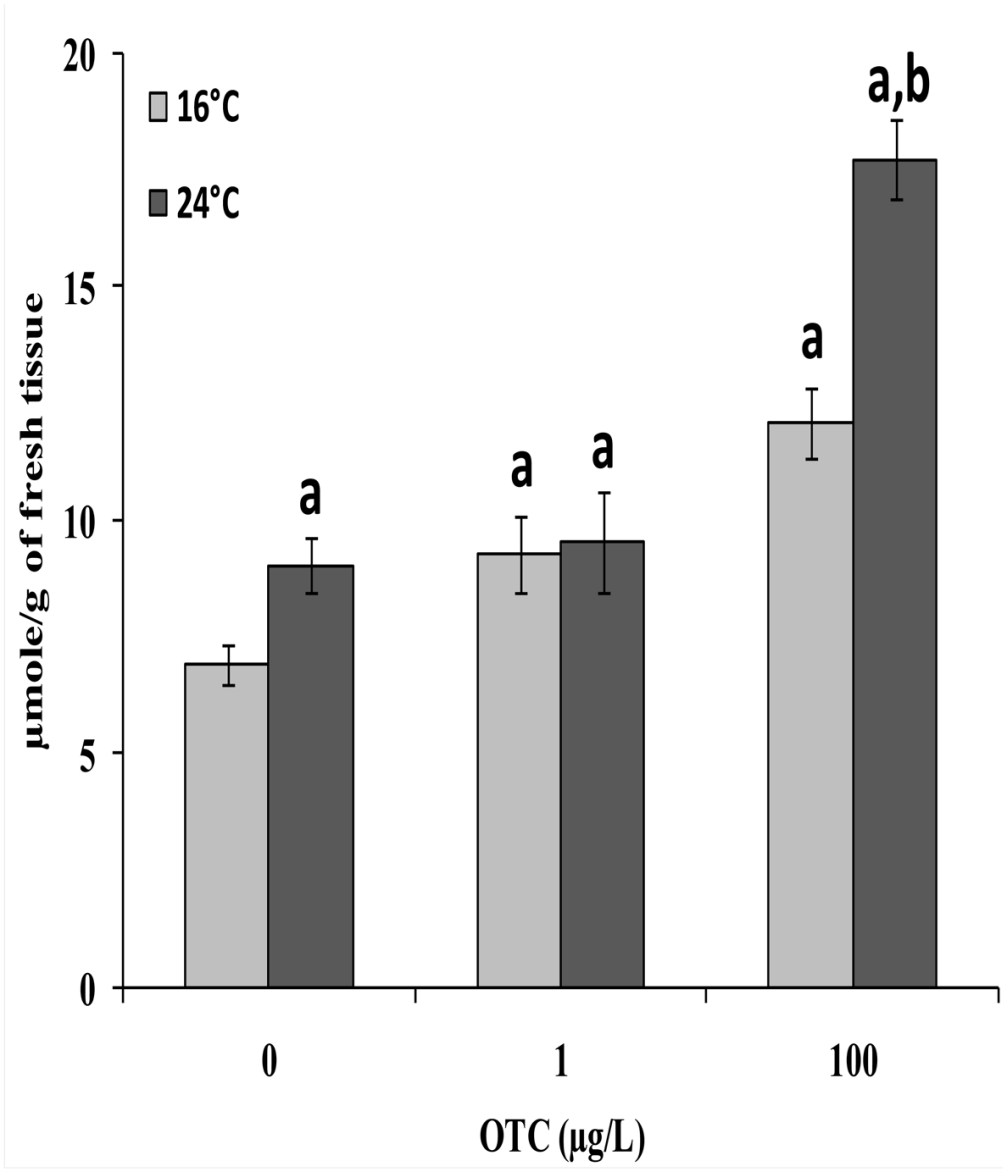
MDA content in gills of mussels after a 4-day treatment with 1 or 100 μg/L tetracycline at 16°C and 24°C. Data are expressed as μmole/g of fresh tissues and represent the mean ± Sd (n = 4). a: significantly different from reference condition. b: significantly different from OTC-untreated mussels at 24°C (p < 0.05).

At 16°C, a moderate but significant increase in MDA was measuredat 1 μg/L OTC, reaching higher values at 100 μg/L. A significant increase in MDA was observed at 24°C compared to 16°C in the absence of OTC, and the MDA content was strongly enhanced at 24°C after treatment with 100 μg/L OTC (Fig 2).

**Fig 3:**
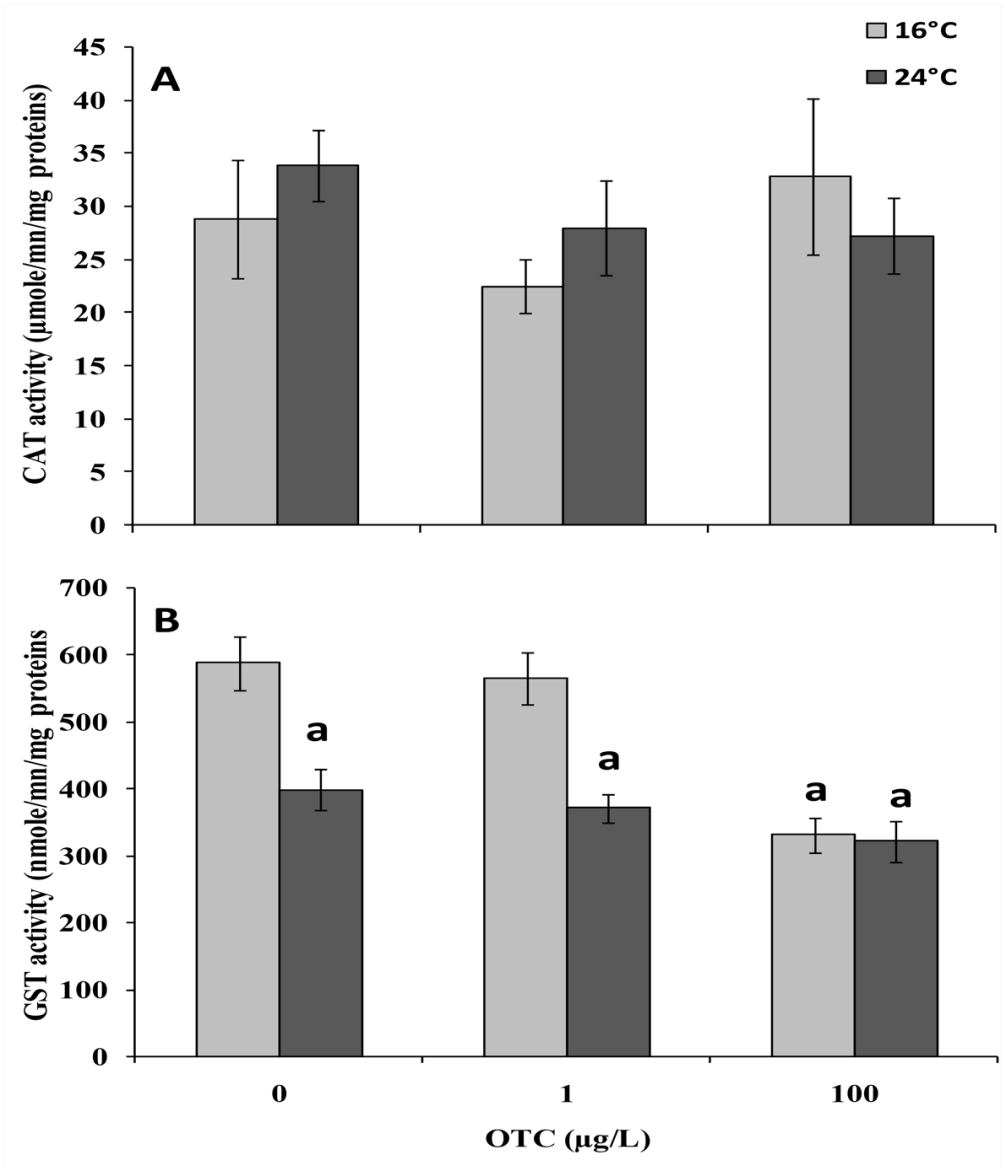
Catalase (CAT; A) and glutathione S-transferase (GST; B) activity in gills of mussels after a 4-day treatment with 1 or 100 μg/L oxytetracycline at 16° and 24°C. Data are expressed as μmole/mn/mg proteins and represent the mean ± Sd (N = 4). a: significantly different from reference condition. b: significantly different from OTC-untreated mussels at 24°C (p < 0.05).

The activities of detoxification enzymes CAT and GST are illustrated in Fig 3. The CAT activity remained unchanged in animals treated with either 1 or 100 μg/L OTC at 16°C and 24°C (Fig 3A). A significant reduction in GST activity was observed in animals exposed to 100 μg/L OTC at 16°C (Fig 3A). Interestingly, GST activity was also significantly reduced with the temperature increase, irrespective of OTC treatment (Fig 3B).

**Fig 4:**
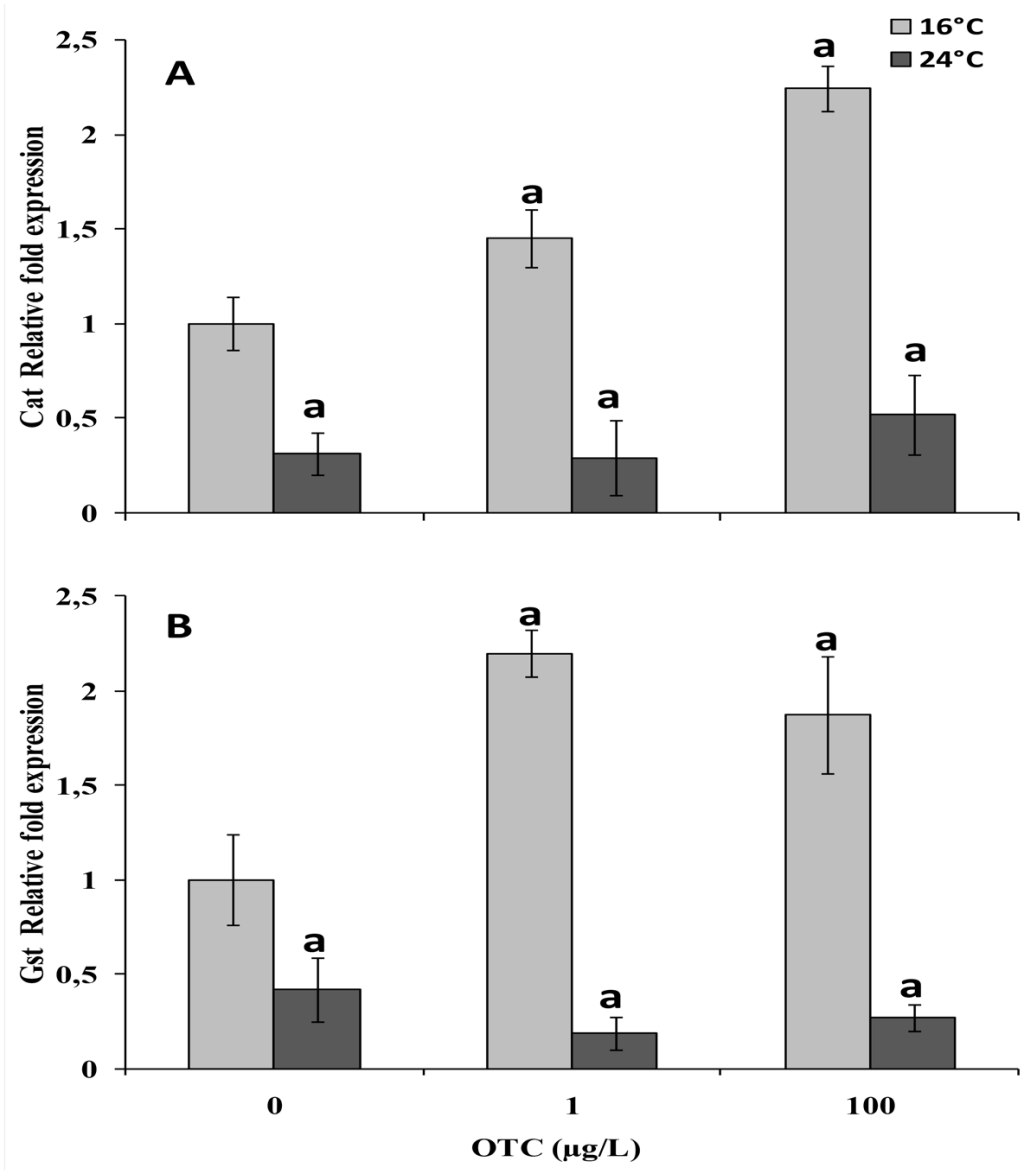
Expression of cat (A) and gst (B) mRNAs in gills of mussels after a 4-day treatment with 1 or 100 μg/L oxytetracycline at 16° and 24°C. Values are expressed as the mean ± SEM (n = 5) of the relative variations (fold change) between each treatment and the reference condition. Data are expressed asnmole/mg proteins and represent the mean ± Sd (n = 4). a: significantly different from reference condition. b: significantly different from OTC-untreated mussels at 24°C (p < 0.05).

A significant increase in cat transcripts was observed at 16°C in gills from OTC-treated animals compared to control animals (Fig 4A). A significant down-regulation of cat transcript was detected in animals maintained at 24°C compared to 16°C, irrespective of the OTC treatment (Fig 4A). At 16°C, OTC treatment (1 or 100 μg/L) increased gst mRNA expression, and a significant down-regulation of gst transcript was detected in animals maintained at 24°C compared to 16°C (Fig 4B), which was further decreased by OTC treatment.

**Fig 5:**
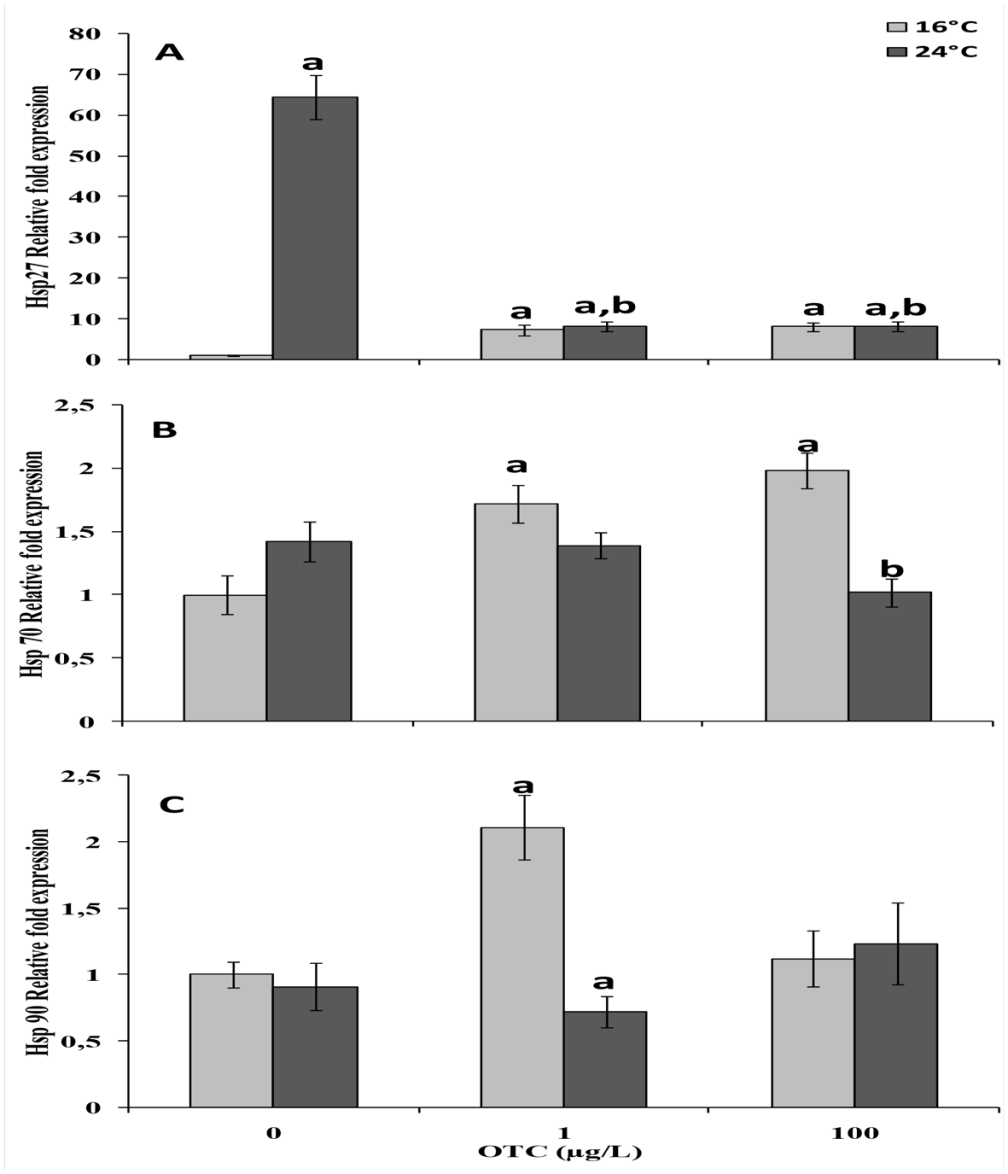
Expression of hsp27 (A), hsp70 (B), and hsp90 (C) mRNA in gills of mussels after a 4-day treatment with 1 or 100 μg/L oxytetracycline at 16° and 24°C. Values are expressed as the mean ± Sd (n = 4) of the relative variations (fold change) between each treatment and the reference condition. a: significantly different from reference condition. b: significantly different from OTC-untreated mussels at 24°C (p < 0.05).

A strong increase (up to 70-fold) of hsp27 mRNA levels was induced in OTC-untreated mussels at 24°C compared to reference mussels at 16°C (Fig 5A). At 16°C, in the presence of OTC, hsp27 transcripts were significantly increased. The effects of treatment with OTC at 24°C shows a drastic reduction in the transcript levels observed at 24°C, but no difference was observed between doses (1 μg/L and 100 μg/L). A significant up-regulation of hsp70 mRNA expression was induced in after treatment with 1 μg/L (~1.75-fold change) and 100 μg/L (~2-fold change) OTC at 16°C compared to control animals (Fig 5B). A significant down-regulation was observed at 24°C at the highest OTC concentration tested. Significant changes in hsp90 transcripts were detected only after treatment with 1 μg/L OTC, with over-expression observed at 16°C and slight down-regulation at 24°C.

**Fig 6:**
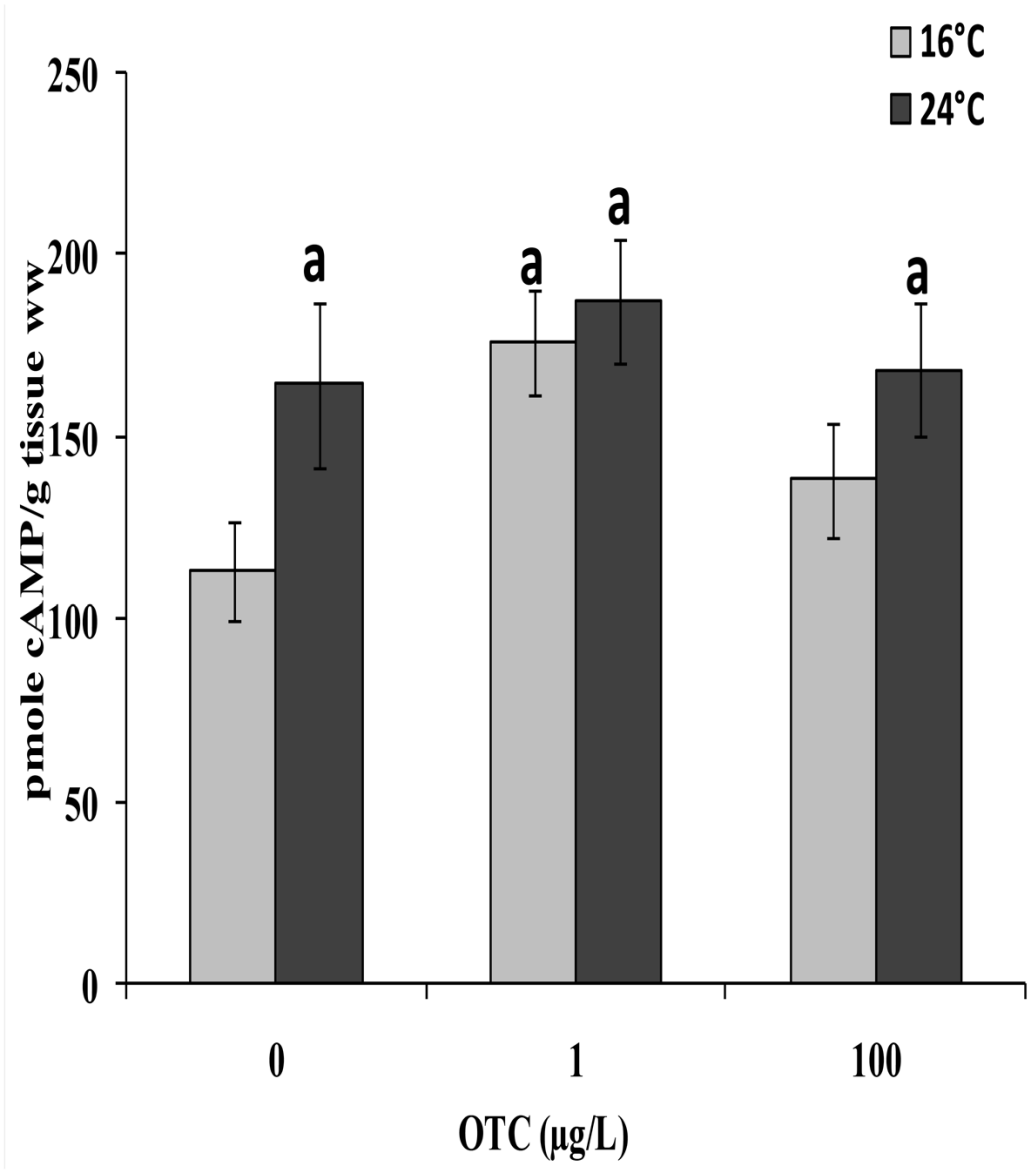
Levels of cAMP in gills of mussels after a 4-day treatment with 1 or 100 μg/L oxytetracycline at 16° and 24°C. Data are expressed as the mean ± Sd (n = 4). a: significantly different from reference condition (p < 0.05).

The cAMP content in gills after a 4-day treatment with OTC (1 μg/L and 100μg/L) is reported in Fig 6. Basal cAMP levels measured in the gills of reference mussels kept at 16°C were 112.23 ± 9.77 pmol cAMP/g tissues. The cAMP levels were significantly increased after treatment with 1 μg/L OTC at 16°C, but no change was observed at 100μg/L OTC with respect to reference mussels. A significant increase was also observed in animals exposed to 24°C, irrespective of OTC treatment.

**Fig 7:**
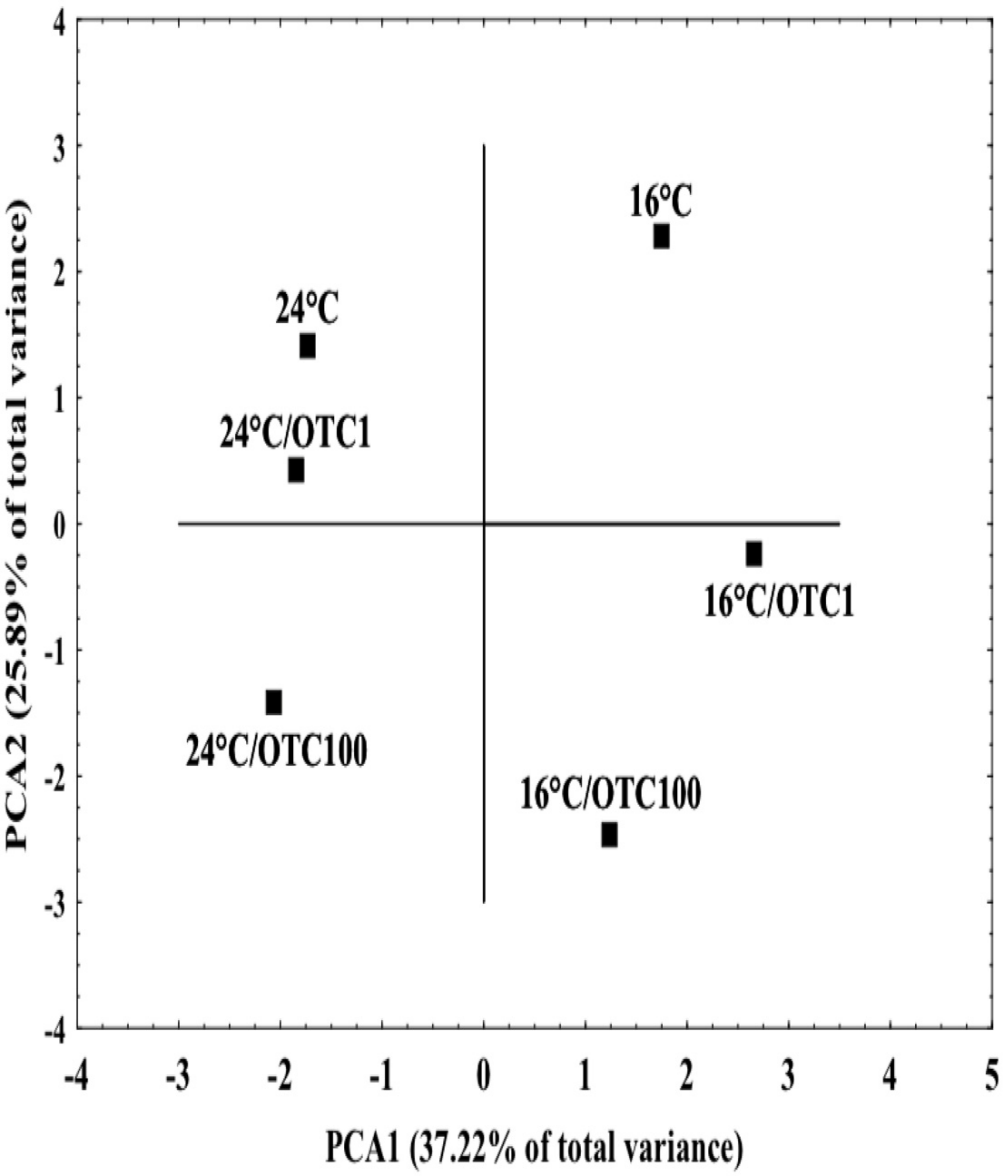
Factor analysis bi-plot of biomarkers (LMS, MDA, CAT activity, GST activity) cell signaling-related parameters (cAMP levels) and mRNA expressions (cat, gst, hsp27, hsp70, hsp90) analyzed in digestive gland and gills of mussels under the different experimental treatments. 16°C: mussels maintained at 16°C and not treated with OTC (reference group); 24°C: mussels maintained at 24°C and not treated with OTC; 16°C/OTC1: mussels maintained at 16°C and treated for 4 days with 1 μg/L OTC; 16°C/OTC100: mussels maintained at 16°C and treated for 4 days with 100 μg/L OTC; 24°C/OTC1: mussels maintained at 24°C and treated for 4 days with 1 μg/L OTC; 24°C/OTC100: mussels maintained at 24°C and treated for 4 days with 100 μg/L OT.

PCAapplied to the biological parameters measured in mussels under the different experimental treatments (Fig 7) showed two principal components (PCA1 and PCA2) explaining 63.1% of the total variance (37.21% and 25.89%, respectively). Variable contribution analyses performed within the PCA indicated that PCA1 is mainly related to the temperature to which mussels were maintained, and PCA2 is mainly associated with OTC treatment. Therefore, temperature accounts for the main effect, with mussels acclimated to 24°C being well separated from those acclimated at 16°C regardless of OTC treatment. At 16°C, OTC-treated mussels were well separated from organisms notexposed to OTC, and significant separation was observed between the two different concentrations of OTC. At 24°C, OTC-exposed groups were less separated from mussels not exposed to OTC.

## Discussion

Climate change encompasses modifications in numerous environmental factors, including pH, water levels, salinity, and temperature [41], and a number of related changes, such as oxygen and food availability, that ultimately modify organism performance and adaptation [42]. In this study, the possible effects of short-term exposure to an emerging contaminant (i.e., the antibiotic OTC) on the molecular and cellular parameters of mussels were assessed at two different temperatures:16°C, typical of the Mediterranean coastal environment, and 24°C, which is considered a possible temperature of the water in the next century and is near the upper border limit for these mollusks. Although scientific research on the impact of global change is an enormously broad field, much can be learned through integrated approaches dealing with the mechanisms underlying biochemical/physiological consequences at the organism scale. Animal responses to environmental changes represent a continuum from the biochemical (i.e., enzyme activation/inhibition and gene expression) and physiological flexibility (i.e., protein expression and metabolic adaptation) that can occur within minutes, hours, or days, to the gene frequency variations and evolutionary changes that alter populations and communities in the long-term.

Temperature is known to affect the physiology of marine invertebrates, and the organisms may become more sensitive to chemical compounds and/or and increase in the pollution level may generate a scenario in which the organisms are less able to cope with the effects of heat stress [24; 25; 26].

OTC represents an emerging contaminant present at concentrations of nano-grams to micrograms per literin coastal environments and may reach a higher level near fish farms. Toxicity tests have reported that OTC induces acute effects, mainly on algae, at concentrations of milligrams per liter [43;44; 45,14]; however, we applied a set of cellular and molecular markers known to respond early to environmental challenges and that are sensitive to pharmaceuticals at environmentally relevant concentrations [27;46]. OTC is rapidly degraded at 16/28°C and at the ionic strength and pH of the marine water used in this study[47]; therefore, OTC levels were not measured. We cannot exclude that the biological effects of this chemical may be partially due to its degradation products. Due to the effects of high temperature and OTC level on oxidative stress in the gills, and because they are the first target of water soluble chemicals, we used gills for the further development of the study.

LMS has been reported to be a very sensitive cellular marker for assessing environmental impact [31;33;48;49]. OTC decreased theLMS in the digestive glands of mussels. The destabilization of lysosomal membranes is an index of increased catabolic activity and chemicals that decrease this parameter also enhance protein catabolism [33] and, more generally, increase the autophagic process in the digestive gland cells [50]. These data have great importance considering that the decrease in LMS is known to correlate with a decrease in the scope for growth (SFG), a biomarker indicating that the energy from the diet is used for growth and reproduction; therefore, a decrease in LMS may be prognostic of negative effects at the population level [31]. Moreover, OTC effects on LMS are more pronounced at 24°C, a temperature that is able to destabilize the lysosomal membranes, which may imply that climate change will exacerbate the effects of OTC on the filter feeding mollusks.

OTC in soil has been reported todecrease the LMS in earthworms [51] with a synergetic effect in presence of Pb and to causes coelomocyte apoptosis.

Moreover, increasing temperature tends to decrease the effect of OTC on LMS in the marine protozoa *Euplotes crassus* [52]. Therefore, the results of the present study highlight the possibility that OTC or its degradation products may have different effects on different organisms depending on its interference with specific metabolic functions of cells/tissues.

Several stressors, including increased temperature, may induce oxidative stress in animals; we assessed biomarkers known to be increased when antioxidant defenses are overwhelmed by ROS production. MDA levels were significantly increased in gills after treatment with OTC. MDA is an intermediate compound formed as a consequence of membrane lipid peroxidation due to oxidative stress. Therefore, the gills appeared to be a target of oxidative stress; indeed, this tissue is the first barrier encountered by soluble pollutants in mussels [53]. Moreover, gills, a highly metabolic active tissue, are often used to evaluate the transcriptomic [25] and proteomic [54] responses of mussels to heat stress and other environmental stressors. Therefore, the detoxification parameters were studied in gill tissue.

Heat stress can affect the physiological status of the animals, often resulting in higher ROS production [55; 56]. Several studies have indicated that tetracyclines may increase oxyradical generation in the cells of exposed organisms [43;57;58]. Like other organisms, mussels can control the increasing ROS levels in their tissues, activating the cellular antioxidative defense systems composed of both enzymatic and non-enzymatic components. The enzymatic pathway consists of protective ROS-scavenging enzymes, such as SOD, CAT, and GST [59;60].

OTC at 1 and 100 μg/L did not significantly change CAT and GST activities, although a decreasing trend was observed for GST at 24°C. Analyses of cat and gst mRNA expression in animals treated with OTC under reference conditions(16°C) showed a marked up-regulation pattern at both concentrations of OTC. Interestingly, a strong decrease in both cat and gst transcripts was observed in animals maintained at 24°C, in the absence or presence of OTC. Therefore, the higher temperature appeared to be able to reduce cat and gst mRNA expression, although the effect was slightly counteracted in the presence of OTC in the case of cat and slightly enhanced in the case of gst. The overall reduction of cat and gst expression at 24°C could reduce mussel capacity to react to thecytotoxic effects of OTC due to the combination of the two stressors.

Toxic chemicals and environmental parameters, such as temperature, may affect the levels of heat shock proteins.[61]. In our laboratories, environmental stress was reported to significantly modify the mRNA expression of various heat shock protein genes [25,26]. Indeed, three clusters of heat shock proteins can be distinguished according to their biological roles. The folding proteins (Hsp70, Hsp90) that play a crucial role inthe restoration of the structure of partially denatured proteins as well as in protein folding and translocation. The small heat shock proteins such as Hsp27, Hsp26 are likely to act as chaperones for maintaining cytoskeletal structural elements during stress (unfolding). Finally, misfoding proteins such as calriticulin known to bind misfolded proteins, preventing their export from the endoplasmic reticulum to the Golgi apparatus [61]. However, to the best of our knowledge, this is the first investigation assessing the heat shock protein response to the presence of OTC at increased temperature, a scenario that may reflect risk to the marine environment in general, and to coastal areas in particular.

Mussel treatment with OTC and exposure to different temperatures affected hsp mRNA expression. In particular, the transcript encoding the small heat shock protein hsp27 was up-regulated in animals exposed to OTC (1 or 100 μg/L) at 16°C. Interestingly, a strong up-regulation was observed in control animals maintained at 24°C; however, in heat-stressed mussels, OTC caused a drastic down-regulation of the mRNA encoding this protein. The small heat shock proteins (hsp27 and hsp26) have been described to play an important role in the response to heat stress [25; 26]. The reduced up-regulation of hsp27 in mussels in the presence of OTC at 24°C compared to those exposed to 24°C in the absence ofOTC may suggest a negative interaction between stressors on the protective hsp27 response in mussels, and in general may underscore an ecological risk due to temperature increases in contaminated seawaters. Moreover, the lack of the small heat shock protein response was reported as the main cause of *Mytilus trossulus*displacement from the Californian coasts in favor of the Mediterranean mussel *Mytilus galloprovincilais* where Hsp27 and Hsp 26 positive regulation was reported as contributor to the invasion success (Lockwood et al., 2010)

Hsp70 mRNA expression was up-regulated in mussels after treatment with OTC at 16°C, anda similar response of hsp27 was observed in animals exposed to 24°C and the highest OTC concentration. This trend confirms a negative interaction between heat stress and OTC exposure; in this situation, mussels may be more prone to the effects of toxic chemicals. Hsp90 mRNA expression was not consistently modulated, with a clear up-regulation only in mussels exposed to 1 μg/L OTC at 16°C. Unfortunately, these latest data do not clarify the physiological significance of this response.

As the heat shock response is partially dependent on cAMP, and changes in cAMP levels in mussel tissues have been reported in the presence of oxidative stress [62;63], metals, pharmaceuticals, and temperature increases [20;45;32], cAMP levels were also evaluated in this study. In general, we observed that cAMP levels are mainly affected by temperature, independent of OTC treatment.

We conclude that the exposure of mussels to environmentally relevant concentrations of OTC deserves attention. The decrease in LMS in the digestive gland of OTC-treated organisms should not be underestimated because this parameter correlates well with the SFG and the health of the animals in the long-term [49]. However, the parameters measured in the present study are also highly affected by incubation of the mussels at 24°C, independent of pharmaceutical administration. High temperature and OTC show a complex pattern of interaction, particularly for the response to oxidative stress. More generally, the temperature effect prevails over OTC treatment. Thus,responseto chemical pollution may be more difficult for the organisms in a changing environment.

## References

[1] Bu Q, Wang B, Huang J, Deng S, Yu G. Pharmaceuticals and personal care products in the aquatic environment in China: A review. J Hazard Mater. 2014; (262): 189–211. 10.1016/j.jhazmat.2013.08.040 24036145

[2] Pickens LB and Tang Y.Oxytetracycline Biosynthesis. JBC. 2010; (285): 27509–27515. 10.1074/jbc.R110.130419 20522541 PMC2934616

[3] Lee HB, Peart TE, Svoboda ML. Determination of endocrine-disrupting phenols, acidic pharmaceuticals, and personal-care products in sewage by solid-phase extraction and gas chromatography-mass spectrometry.J chromatogra A. 2005;1094(1–2):122–9. 10.1016/j.chroma.2005.07.070 16257298

[4] Vieno NM, Tuhkanen T, Kronberg L.Analysis of neutral and basic pharmaceuticals in sewage treatment plants and in recipient rivers using solid phase extraction and liquid chromatography-tandem mass spectrometry detection. J chromatogra A. 2006;1134(1–2):101–11. 10.1016/j.chroma.2006.08.077 16996072

[5] Calamari D, Zuccato E, Castiglioni S, Bagnati R, Fanelli R. Strategic survey of therapeutic drugs in the rivers Po and Lambro in northern Italy. Environ Sci Technol. 2003; 37 (7): 1241–1248.

[6] Weigel S, Kuhlmann J, Huhnerfuss H. Drugs and personal care products as ubiquitous pollutants: occurrence and distribution of clofibric acid, caffeine and DEET in the North Sea. Sci Total Environ. 2002;295 (1–3): 131–141. 12186282 10.1016/s0048-9697(02)00064-5

[7] Fent K, Weston AA, Caminada A. Ecotoxicology of human pharmaceuticals.Aqua Tox. 2006; 76:122–159. 10.1016/j.aquatox.2005.09.009 16257063

[8] Santos LH, Araújo AN, Fachini A, Pena A, Delerue-Matos C, Montenegro MC. Ecotoxicological aspects related to the presence of pharmaceuticals in the aquatic environment.J Hazard Mater. 2010 175(1–3): 45–95. 19954887 10.1016/j.jhazmat.2009.10.100

[9] Crane M, Watts C, Boucard T. Chronic aquatic environmental risks from exposure to human pharmaceuticals. Sci Total Environ. 2006; 367(1), 23–41. 16762401 10.1016/j.scitotenv.2006.04.010

[10] Na G, Fang X, Cai Y, Ge L, Zong H, Yuan X et al Occurrence, distribution, and bioaccumulation of antibiotics in coastal environment of Dalian, China. Mar Pollut Bull.2013; 69(1–2), 233–237. 10.1016/j.marpolbul.2013.01.020 23465572

[11] Kołodziejska M, Maszkowska J, Białk-Bielińska A, Steudte S, Kumirska J, Stepnowski et al Aquatic toxicity of four veterinary drugs commonly applied in fish farming and animal husbandry. Chemosphere. 2013; 92(9), 1253–9. 10.1016/j.chemosphere.2013.04.057 23689096

[12] Reemtsma T, Jekel M: Organic pollutants in the water cycle. Weinheim: Wiley 2006; 341pp.

[13] Kolar B, Arnuš L, Jeretin B, Gutmaher A, Drobne D, Durjava MK.The toxic effect of oxytetracycline and trimethoprim in the aquatic environment. Chemosphere. 2014; 115,75–80. 10.1016/j.chemosphere.2014.02.049 24703011

[14] Feng-Jiao L, Shun-Xing L, Feng-Ying Z, Xu-Guang H, Yue-Gang Z, Teng-Xiu T, et al Risk assessment of nitrate and oxytetracycline addition on coastal ecosystem functions. Aqua Tox. 2014 146 (2014) 76–81. 10.1016/j.aquatox.2013.10.028 24287139

[15] Carere M, Dulio V, Hanke G, Polesello S. Guidance for sediment and biota monitoring under the common implementation strategy for the water framework directive. Trends Analyt Chem. 2012; (36), 15–24

[16] Helmuth B, Harley CD, Halpin PM, O'Donnell M, Hofmann GE, Blanchette CA. Climate change and latitudinal patterns of intertidal thermal stress. Science. 2002; 298(5595), 1015–7. 12411702 10.1126/science.1076814

[17] Smit CE and Van Gestel CA. Influence of temperature on the regulation and toxicity of zinc in *Folsomia candida* (Collembola). EcotoxicolEnviron Saf. 1997; 37(3), 213–22. 9378087 10.1006/eesa.1997.1558

[18] Angilletta MJ, Sears MW, Pringle RM. Spatial dynamics of nesting behavior: lizards shift microhabitats to construct nests with beneficial thermal properties. Ecology. 2009; 90(10), 2933–9. 19886501 10.1890/08-2224.1

[19] Pörtner HO, Farrell AP. Ecology, Physiology and climate change. Science. 2008; 31;322(5902), 690–2. 10.1126/science.1163156 18974339

[20] Fabbri E, Chen X, Capuzzo A, Moon TW. Binding kinetics and sequencing of hepatic alpha1-adrenergic receptors in two marine teleosts, mackerel (*Scomber scombrus*) and anchovy (*Engraulis encrasicolus*). J Exp Zool A Ecol Genet Physiol. 2008; 309(3), 157–65. 10.1002/jez.441 18273865

[21] Piano A, Asirelli C, Caselli F, Fabbri E. Hsp70 expression in thermally stressed Ostrea edulis, a commercially important oyster in Europe. Cell Stress Chaperones. 2002; 7(3), 250–7. 12482201 10.1379/1466-1268(2002)007<0250:heitso>2.0.co;2 PMC514825

[22] Vergani L, Grattarola M, Borghi C, Dondero F, Viarengo A. Fish and molluscan metallothioneins. FEBS J. 2005; 272(23):6014–23. 16302966 10.1111/j.1742-4658.2005.04993.x

[23] Somero GN. The physiology of global change: linking patterns to mechanisms. Ann Rev Mar Sci. 2012; (4), 39–61. 22457968 10.1146/annurev-marine-120710-100935

[24] Kamel N, Attig H, Dagnino A, Boussetta H, Banni M. Increased temperatures affect oxidative stress markers and detoxification response to benzo[a]pyrene exposure in mussel *Mytilus galloprovincialis* . Arch Environ Contam Toxicol. 2012; 63(4),534–43. 10.1007/s00244-012-9790-3 22903631

[25] Negri A, Oliveri C, Sforzini S, Mignione F, Viarengo A, Banni M.Transcriptional response of the mussel Mytilus galloprovincialis (Lam.) following exposure to heat stress and copper. PLoS One. 2013; 8(6), e66802 10.1371/journal.pone.0066802 23825565 PMC3692493

[26] Banni M, Hajer A, Sforzini S, Oliveri C, Boussetta H, Viarengo A.Transcriptional expression levels and biochemical markers of oxidative stress in *Mytilus galloprovincialis* exposed to nickel and heat stress. Comp Biochem Physiol C Toxicol Pharmacol. 2014; (160), 23–9. 10.1016/j.cbpc.2013.11.005 24291086

[27] Franzellitti S and Fabbri E. Cyclic-AMP Mediated Regulation of ABCB mRNA Expression in Mussel Haemocytes.PLoS One.2013; 8(4): e61634 10.1371/journal.pone.0061634 23593491 PMC3625153

[28] Banni M, Negri A, Rebelo M, Rapallo F, Boussetta H. Expression analysis of the molluscan p53 protein family mRNA in mussels (*Mytilus* spp.) exposed to organic contaminants. Comp Biochem Physiol C Toxicol Pharmacol.2009; 149, 414–418. 10.1016/j.cbpc.2008.09.017 18973830

[29] Canesi L, Negri A, Barmo C, Banni M, Gallo G, Viarengo A et al The organophosphate chlorpyrifos interferes with the responses to 17b-estradiol in the digestive gland of the marine mussel *Mytilus galloprovinciali* . PLos One. 2011; 6 (5),e19803 10.1371/journal.pone.0019803 21625485 PMC3098840

[30] Franzellitti S, Viarengo A, Dinelli E, Fabbri E. Molecular and cellular effects induced by hexavalent chromium in Mediterranean mussels. Aquat Toxicol. 2012 (15), 125–32. 10.1016/j.aquatox.2012.07.011 22940607

[31] Dondero F, Banni M, Negri A, Boatti L, Dagnino A, Viarengo A. Interactions of a pesticide/heavy metal mixture in marine bivalves: a transcriptomic assessment. BMC Genomics. 2011; 12: 195 10.1186/1471-2164-12-195 21496282 PMC3094310

[32] Fabbri E., Capuzzo A. 2010. Cyclic AMP signaling in bivalve molluscs: an overview. J Exp Zool A Ecol Genet Physiol. 2010; 313(4): 179–200. 10.1002/jez.592 20127660

[33] Viarengo A, Lowe D, Bolognesi C, Fabbri E, Koehler A. The use of biomark-ers in biomonitoring: a 2-tier approach assessing the level of pollutant-inducedstress syndrome in sentinel organisms. Comp Biochem Physiol C Toxicol Pharmacol. 2007; 146 (3), 281–300. 17560835 10.1016/j.cbpc.2007.04.011

[34] Moore MN and Viarengo A. Lysosomal membrane fragility and catabolismof cytosolic proteins: evidence for a direct relationship. Experientia. 1987; 43 (3),320–323. 3556528 10.1007/BF01945568

[35] Gérard-Monnier D, Erdelmeier I, Régnard K, Moze-Henry N, Yadan J, Chaudière J. Reactions of 1-methyl-2-phenylindole with malondialde-hyde and 4-hydroxyalkenals. Analytical applications to a colorimetric assay of lipid peroxidation. Chem Res Toxicol. 1989; 11 (10), 1176–1183. 10.1021/tx9701790 9778314

[36] Mimeault C, Trudeau VL, Moon TW. Waterborne gemfibrozil challenges the hepatic antioxidant defense system and down-regulates peroxisome proliferator-activated receptor beta (PPARbeta) mRNA levels in male goldfish (*Carassius auratus*). Toxicology. 2006; 7;228(2–3), 140–50. 17046140 10.1016/j.tox.2006.08.025

[37] Lowry OH, Rosebrough NJ, Farr AL, Randall RJ. Protein measurement with the Folin phenol reagent. JBC. 1951; 193, 265–275. 14907713

[38] Chomczynski P and Sacchi N. 1987. Single-step method of RNA isolation by acid guanidinium thiocyanate–phenol–chloroform extraction. Anal Biochem. 1987; 162, 156–169. 2440339 10.1006/abio.1987.9999

[39] Banni M, Negri A, Mignone F, Boussetta H, Viarengo A, Dondero F.Gene expression rhythms in the mussel *Mytilus galloprovincialis* (Lam.) across an annual cycle. PLoSOne. 2011; 6 (5), e18904 10.1371/journal.pone.0018904 21573210 PMC3088662

[40] Pfaffl MW, Horgan GW, Dempfle L. Relative expression software tool (REST) for group wise comparison and statistical analysis of relative expression results in real-time PCR. Nucleic Acids Res. 2002; 30, e36 11972351 10.1093/nar/30.9.e36 PMC113859

[41] Helmuth B. From cells to coastlines: how can we use physiology to forecast the impacts of climate change? J Exp Biol. 2009; 212(6), 753–60. 10.1242/jeb.023861 19251989

[42] Monaco CJ and Helmuth B. Tipping points, thresholds and the keystone role of physiology in marine climate change research.Adv Mar Biol. 2011; 60, 123–60. 10.1016/B978-0-12-385529-9.00003-2 21962751

[43] Elia AC, Ciccotelli V, Pacini N, Dörr AJ, Gili M, Natali M, et al Transferability of oxytetracycline (OTC) from feed to carp muscle and evaluation of the antibiotic effects on antioxidant systems in liver and kidney. Fish Physiol Biochem. 2014;40(4), 1055–68. 10.1007/s10695-013-9905-4 24390127

[44] Gagné F, Blaise C, Fournier M, Hansen PD. Effects of selected pharmaceutical products on phagocytic activity in *Elliptio complanata* mussels. Comp Biochem Physiol C Toxicol Pharmacol. 2006; 143, 179–186. 16533621 10.1016/j.cbpc.2006.01.008

[45] Franzellitti S., Buratti S., Valbonesi P., Capuzzo A., Fabbri E. The β-blocker propranolol affects cAMP-dependent signaling and induces the stress response in Mediterranean mussels, *Mytilus galloprovincialis* . Aquat Toxicol. 2011; (101): 299–308. 10.1016/j.aquatox.2010.11.001 21216339

[46] Gust M, Gélinas M, Fortier M, Fournier M, Gagné F.In vitro immunotoxicity of environmentally representative antibiotics to the freshwater mussel *Elliptio complanata* . Environ Pollut. 2012; 169, 50e58. 22683480 10.1016/j.envpol.2012.05.020

[47] Ratasuk N, Boonsaner M, Hawker DW. Effect of temperature, pH and illumination on abiotic degradation of oxytetracycline in sterilized swine manure. J Environ Sci Health A Tox Hazard Subst Environ Eng.2012; 47(11):1687–94. 10.1080/10934529.2012.687274 22702830

[48] Allen JI, Moore MN. Environmental prognostics: is the current use of biomarkers appropriate for environmental risk evaluation? Mar Environ Res. 2004; 58(2–5), 227–32. 15178036 10.1016/j.marenvres.2004.03.119

[49] McVeigh A, Allen JI, Moore MN, Dyke P, Noble D. A carbon and nitrogen flux model of mussel digestive gland epithelial cells and their simulated response to pollutants. Mar Environ Res. 2004; 58(2–5),821–27. 15178119 10.1016/j.marenvres.2004.03.099

[50] Moore MN, Kohler A, Lowe D, Viarengo A. Lysosomes and autophagy in aquatic animals. Methods Enzymol. 2008; (451):581–620. 10.1016/S0076-6879(08)03233-3 19185741

[51] Gao M, Zhou Q, Song W, Ma X. Combined effects of oxytetracycline and Pb on earthworm *Eisenia* fetida. Environ toxicol pharmacol. 2014; 37(2):689–96. 10.1016/j.etap.2014.02.004 24607684

[52] Gomiero A, Viarengo A. Effects of elevated temperature on the toxicity of copper and oxytetracycline in the marine model, *Euplotes crassus*: A climate change perspective. Environ Pollut.2014; (194): 262–71. 10.1016/j.envpol.2014.07.035 25163430

[53] Vidal-Liñán L and Bellas J. Practical procedures for selected biomarkers in mussels, Mytilus galloprovincialis--implications for marine pollution monitoring. Sci Total Environ. 2013; (461–462): 56–64. 10.1016/j.scitotenv.2013.04.079 23712116

[54] Tomanek L, Zuzow MJ. The proteomic response of the mussel congeners *Mytilus galloprovincialis* and *M*. *trossulus* to acute heat stress: implications for thermal tolerance limits and metabolic costs of thermal stress. J Exp Biol. 2010; 213 (Pt 20): 3559–3574. 10.1242/jeb.041228 20889836

[55] Rajagopal S, Van der Velde G, Van der Gaag M, Jenner HA.Factors influencing the upper temperature tolerances of three mussel species in a brackish water canal: size, season and laboratory protocols. Biofouling. 2005; 21 (2), 87–97. 16167389 10.1080/08927010500133584

[56] Verlecar XN, Jena KB, Chainy GB. Biochemical markers of oxidative stressin Perna viridis exposed to mercury and temperature. Chem Biol Interact. 2007; 167 (3): 219–226. 17418111 10.1016/j.cbi.2007.01.018

[57] Petrenko IU, Titov V, Vladimirov IA. Generation of active forms of oxygen by antibiotics of the tetracycline series during tetracycline catalysis of oxidation of ferrous iron. Antibiot Khimioter. 1995; 40(2), 3–8. 7605140

[58] Quinlan GJ, Gutteridge JM Hydroxyl radical generation by the tetracycline antibiotics with free radical damage to DNA, lipids and carbohydrate in the presence of iron and copper salts. Free Radic Biol Med. 1988; 5(5–6): 341–8. 2855734 10.1016/0891-5849(88)90106-2

[59] Atli G, Alptekin O, Tukel S, Canli M. Response of catalase activity to Ag2+, Cd^2+^, Cr2+, Cu^2+^ and Zn^2+^ in five tissues of freshwater fish *Oreochromis niloticus* . Comp Biochem Physiol. C. 2006;(143): 218–224. 10.1016/j.cbpc.2006.02.003 16581305

[60] Feder ME, Hofmann GE. Heat-shock proteins, molecular chaperones, and the stress response: evolutionary and ecological physiology.Annu Rev Physiol. 1999; (61): 243–82. 10099689 10.1146/annurev.physiol.61.1.243

[61] Ruas CBG, Carvalho CdS, De Araujo HSS, Espindola ELG, Fernandes MN.Oxidative stress biomarkers of exposure in the blood of cichlid species from a metal-contaminated river. Ecotoxicol Environ Saf. 2008; 71(1):86–93. 17936357 10.1016/j.ecoenv.2007.08.018

[62] Koutsogiannaki S, Franzellitti S, Fabbri E, Kaloyianni M. Oxidative stress parameters induced by exposure to either cadmium or 17β-estradiol on *Mytilus galloprovincialis* hemocytes. The role of signaling molecules. Aquat Toxicol. 2014; 146,186–95. 10.1016/j.aquatox.2013.11.005 24316436

[63] Raftopoulou M. Protein expression: one by one. Nature cell biology. 2006; 8(5):429 16691205 10.1038/ncb0506-429

